# Impact of growth mindset on college students’ career decision-making self-efficacy: the chain-mediating roles of perceived social support and meaning in life

**DOI:** 10.3389/fpsyg.2026.1710494

**Published:** 2026-02-04

**Authors:** Song Liu, Zhilin Ping, Chongyuan Fan, Yanzhi Mo, Yixuan Wang

**Affiliations:** 1 School of Marxism, Hunan University of Chinese Medicine, Changsha, Hunan, China; 2 School of Marxism, Hunan Academy of Governance, Changsha, Hunan, China; 3 School of Humanities and Management, Hunan University of Chinese Medicine, Changsha, Hunan, China; 4 Department of Psychology, Hunan University of Chinese Medicine, Changsha, Hunan, China; 5 School of Education, Soochow University, Suzhou, Jiangsu, China

**Keywords:** career decision-making self-efficacy, college students, growth mindset, meaning in life, perceived social support

## Abstract

**Background:**

In the post-pandemic era, the number of college graduates has continued to reach new highs, while employment rates have fallen to a ten-year low. Career decision self-efficacy (CDSE) has been shown to significantly predict job search persistence and employment quality; however, the underlying mechanisms remain unclear. Growth mindset is considered an intervenable psychological resource, and its role in areas such as health and academics has been demonstrated. However, empirical research explaining how it is transformed into career confidence through the chain pathway of “perceived social support → meaning in life” is lacking. Clarifying this progressive mechanism could provide evidence-based strategies for university career guidance.

**Objective:**

The study aimed to examine the chain-mediated effects of perceived social support and meaning in life on the relationship between growth mindset and career decision-making self-efficacy among college students.

**Method:**

A survey was conducted among 556 students from Hunan University of Traditional Chinese Medicine using the Growth Mindset Scale (GMS), the Career Decision Self-Efficacy Scale–Short Form (CDSE-SF), the Multidimensional Scale of Perceived Social Support (MSPSS), and the Chinese version of the Meaning in Life Questionnaire (C-MLQ).

**Results:**

Correlation analysis showed significant positive correlations between growth mindset, career decision-making self-efficacy, perceived social support, and meaning in life (r = 0.359–0.538, *p* < 0.001). Growth mindset had a significant direct effect on career decision-making self-efficacy (*β* = 1.761). Growth mindset also positively predicted perceived social support (*β* = 1.278) and meaning in life (*β* = 0.978). Perceived social support positively predicted meaning in life (*β* = 0.017) and career decision-making self-efficacy (*β* = 0.433). Moreover, meaning in life positively predicted career decision-making self-efficacy (*β* = 0.304). Bootstrap analysis confirmed that the chain-mediated effect of perceived social support and meaning in life on the relationship between growth mindset and career decision-making self-efficacy was significant, accounting for 34.24% of the total effect. The effect values of the three mediation pathways accounted for 20.65, 2.46, and 11.13% of the total effect.

**Conclusion:**

Growth mindset can directly enhance college students’ career decision-making self-efficacy and can also indirectly influence it through perceived social support and meaning in life.

## 1 Introduction

With the number of college graduates reaching a record high nationwide, competition in the job market for college students has intensified. Career decision-making self-efficacy refers to an individual’s confidence in their ability to engage in career selection and planning (Li et al., 2022). Individuals with higher levels of career decision-making self-efficacy are better able to evaluate their strengths and skills and make informed career choices, which is conducive to employment success (Anyango et al., 2024). Therefore, examining college students’ career decision-making self-efficacy and its influencing factors has important implications in alleviating employment pressure and improving overall employment rates.

Growth mindset refers to individuals’ cognitions and beliefs about their own abilities, specifically the belief that ability and intelligence can be continuously improved through sustained effort and learning (Dweck, 2006). Individuals with a stronger growth mindset typically exhibit greater adaptability and are less likely to withdraw in the face of failure; instead, they tend to actively confront new challenges and opportunities (Wolcott et al., 2021). The career decision-making process is characterized by uncertainty and complexity. A growth mindset can help college students adopt positive coping strategies when facing confusion or challenges in their career choices. Based on this, Hypothesis 1 is proposed: Growth mindset directly affects career decision-making self-efficacy.

Perceived social support refers to an individual’s subjective perception of support from close relationships. It reflects their desire to be understood, respected, and cared for, as well as their cognitive evaluation of social connections and emotional support (Uchino et al., 2022). The growth mindset of college students has been shown to predict career decision-making self-efficacy and can also indirectly influence it through perceived social support and meaning in life (Pignault et al., 2023). Previous research indicates that higher levels of perceived social support are associated with stronger career decision-making self-efficacy (Murry et al., 2024). Accordingly, Hypothesis 2 is proposed: Growth mindset affects career decision-making self-efficacy through the mediating effect of perceived social support.

According to Steger, meaning in life refers to an individual’s deep understanding of their own life, as well as their awareness of personal goals, missions, or important life tasks (Steger et al., 2008). Two recent longitudinal studies further showed that growth mindset not only enhances academic or career performance in the short term but also enables individuals to experience higher levels of meaning in life through a “challenge–reflection–value reconstruction” pathway when facing adversity (Rush et al., 2021; Zhao et al., 2023). In other words, individuals with a growth mindset are more likely to view setbacks as opportunities for self-growth, thereby endowing life with renewed purpose. It can be inferred that the influence of growth mindset on career decision-making self-efficacy is at least partially realized through the process in which it first enhances meaning in life and then strengthens confidence. Therefore, Hypothesis 3 is proposed: Growth mindset positively influences career decision-making self-efficacy through the mediating role of meaning in life.

Studies have shown that perceived social support is closely related to meaning in life (Zhang et al., 2025). Social support can provide emotional comfort and a sense of belonging, helping individuals recognize their values and importance within society, thereby enhancing their sense of meaning in life. Individuals with higher perceived social support tend to be more confident when facing life challenges and have a clearer sense of life purpose and meaning. Integrating the above hypotheses, college students with a high level of growth mindset are more likely to receive support from others when facing challenges, which, in turn, enhances their understanding and perception of meaning in life and ultimately improves their confidence and self-efficacy in career decision-making. Based on the resource–meaning–action framework, growth mindset first enhances perceptions of social support (external resources), which then strengthens meaning in life (internal meaning framework), thereby improving career decision-making self-efficacy (goal-directed action). Accordingly, Hypothesis 4 is proposed: Growth mindset indirectly affects career decision-making self-efficacy through perceived social support and meaning in life, with these two factors playing chain-mediating roles.

In summary, this study collected data through an online questionnaire to explore the influencing factors and mechanisms underlying college students’ career decision-making self-efficacy, with a particular focus on the chain-mediating roles of perceived social support and meaning in life in the relationship between growth mindset and career decision-making self-efficacy.

## 2 Materials and methods

### 2.1 Participants

Owing to limitations in time, funding, and human resources, focusing on a single university allowed for more efficient data collection and analysis, thereby enhancing the feasibility of the study. Therefore, a survey was conducted among undergraduate students at Hunan University of Chinese Medicine using electronic questionnaires. Ethical approval was obtained from the university’s ethics committee, and informed consent was obtained from all participants.

The inclusion criteria were full-time undergraduate students at Hunan University of Chinese Medicine aged 18–25 years. The exclusion criteria included part-time students, postgraduate students, and students who had withdrawn from the university.

### 2.2 Tools

#### 2.2.1 Growth Mindset Scale (GMS)

The Growth Mindset Scale (GMS) (Dweck, 2006) consists of two dimensions—growth mindset and fixed mindset—and includes six items. Each item is scored on a 6-point Likert scale ranging from 1 (completely inconsistent) to 6 (completely consistent). The total scores range from 6 to 36, with higher scores indicating a stronger growth mindset. Cronbach’s *α* was 0.876, indicating excellent internal consistency.

#### 2.2.2 Career Decision Self-Efficacy Scale–Short Form (CDSE-SF)

The Career Decision Self-Efficacy Scale–Short Form (CDSE-SF) (Peng and Long, 2001) comprises five dimensions—self-evaluation, information collection, goal selection, planning, and problem-solving—and includes 39 items. Each item is rated on a 5-point Likert scale ranging from 1 (not at all confident) to 5 (completely confident). The total scores range from 39 to 195, with higher scores indicating higher levels of career decision-making self-efficacy. Cronbach’s *α* was 0.854, indicating good reliability.

#### 2.2.3 Multidimensional Scale of Perceived Social Support (MSPSS)

The Multidimensional Scale of Perceived Social Support (MSPSS) (Zimet et al., 1990) consists of three dimensions—family support, friend support, and other support—and includes 12 items. Each item is rated on a 7-point Likert scale ranging from 1 (strongly disagree) to 7 (strongly agree). The total scores range from 12 to 84, with higher scores indicating higher perceived social support. Cronbach’s *α* was 0.906, indicating very good reliability.

#### 2.2.4 Chinese version of the Meaning in Life Questionnaire (C-MLQ)

The Chinese version of the Meaning in Life Questionnaire (C-MLQ) (Chen and Gao, 2021) includes two dimensions—presence of meaning in life and search for meaning in life—and comprises 10 items. Each item is rated on a 7-point Likert scale ranging from 1 (strongly disagree) to 7 (strongly agree). The total scores range from 10 to 70, with higher scores indicating a stronger sense of meaning in life. Cronbach’s α was 0.880, indicating excellent reliability.

### 2.3 Data collection process

A survey was conducted among undergraduate students at Hunan University of Chinese Medicine using electronic questionnaires. During the questionnaire design stage, a survey instrument was developed that included several sections (general demographic information, the GMS, CDSE-SF, MSPSS, and C-MLQ). The questionnaire underwent expert review and pre-testing to ensure validity and reliability. The questionnaires were distributed through the university’s official channels, including promotional posters with QR codes placed in public areas such as bulletin boards, student activity centers, and libraries, allowing students to easily scan and complete the survey. In addition, questionnaire links and instructions were sent to all the enrolled students via the university’s official email system to encourage participation.

Participants’ personal information and responses were kept strictly confidential and were used solely for research purposes. The data were processed anonymously, and no personally identifiable information was disclosed. In total, 569 students completed the questionnaire. During data cleaning, 13 questionnaires were excluded owing to incomplete or missing key data (e.g., unanswered key questions or clearly inconsistent responses). Ultimately, 556 valid questionnaires were retained, yielding a response rate of 97.7%.

### 2.4 Sample size calculation

The sample size was determined using statistical methods. Based on the estimated population size (approximately 4,000 students at Hunan University of Chinese Medicine), a confidence level of 95%, and an allowable error of 5%, the standard sample size calculation formula $n=\frac{Z^{2}\times p(1-p)}{E^{2}}$ was applied, where Z represents the Z value corresponding to a 95% confidence level (approximately 1.96), p is the estimated proportion (set at 0.5 to maximize the sample size), and E represents the allowable error (0.05). The minimum required sample size was calculated to be greater than 350. The 556 valid questionnaires collected exceeded this requirement, ensuring the reliability and representativeness of the study results.

### 2.5 Statistical methods

Statistical analyses were performed using SPSS version 25.0. Data were collected using a questionnaire-based approach, and common method bias was assessed using Harman’s single factor test. The SPSS PROCESS macro was used to test the multiple mediation model (Model 6). Statistical significance was set at a *p*-value of < 0.05.

## 3 Results

### 3.1 Baseline data of the participants

The mean age of the 556 participants was 21.55 ± 2.05 years. The sample comprised 310 male students (55.76%) and 246 female students (44.24%). The number of freshmen, sophomores, juniors, and seniors was 122 (21.94%), 163 (29.32%), 148 (26.62%), and 123 (22.12%), respectively. Detailed characteristics of the study participants are listed in Table 1.

**Table 1:** Baseline data of the study participants.

Variable	*n* (%) / Mean ± SD
Age (years)	21.55 ± 2.05
Sex	
Male	310 (55.76)
Female	246 (44.24)
Grade level	
First grade	122 (21.94)
Second grade	163 (29.32)
Third grade	148 (26.62)
Fourth grade	123 (22.12)
Source location	
Rural	324 (58.27)
Town	232 (41.73)
The only child	
Yes	143 (25.72)
No	413 (74.28)
Monthly household income (yuan)	
<5,000	164 (29.50)
5,000-100,00	291 (52.34)
>10,000	101 (18.17)

### 3.2 Scores of the different scales

Among the 556 participants in this study, the total scores of the GMS, CDSE-SF, MSPSS, and C-MLQ were 27.51 ± 3.27, 114.95 ± 20.28, 60.92 ± 9.59, and 46.39 ± 7.73, respectively. Table 2 presents the dimensions and total scores of the different scales.

**Table 2:** Scores of the different scales.

Scale	Dimensionality	Score	Skewness		Kurtosis	
			Value	S.E.	Value	S.E.
Growth mindset scale	Growth thinking	14.06 ± 1.60	−0.604	0.104	0.403	0.207
	Fixed thinking	13.45 ± 2.25	0.065	0.104	−1.152	0.207
	Total	27.51 ± 3.27	−0.294	0.104	−0.157	0.207
Career decision self-efficacy scale–short form	Self-evaluation	15.19 ± 4.80	0.911	0.104	−0.473	0.207
	Information collection	29.42 ± 5.69	−1.227	0.104	1.339	0.207
	Goal selection	31.40 ± 4.84	−0.853	0.104	0.809	0.207
	Planning	17.65 ± 5.70	1.282	0.104	0.984	0.207
	Problem solving	21.28 ± 7.21	−0.51	0.104	−1.178	0.207
	Total	114.95 ± 20.28	0.153	0.104	−0.455	0.207
Multidimensional scale of perceived social support	Family support	21.57 ± 3.24	−0.093	0.104	−0.824	0.207
	Friend support	19.96 ± 3.07	−0.374	0.104	0.135	0.207
	Other support	19.39 ± 5.00	−0.896	0.104	−0.388	0.207
	Total	60.92 ± 9.59	−0.446	0.104	−0.475	0.207
The Chinese version of the meaning in life questionnaire	Presence of meaning in life	21.20 ± 4.13	0.525	0.104	−0.594	0.207
	Search for meaning in life	25.19 ± 4.98	−0.865	0.104	−0.271	0.207
	Total	46.39 ± 7.73	−0.149	0.104	−0.691	0.207

### 3.3 Common method bias test

To examine the potential influence of common method bias, Harman’s single factor test was conducted. As shown in Table 3, seven factors had eigenvalues greater than 1, and the first factor accounted for 31.07% of the total variance, indicating that the results of this study were not significantly affected by common method bias.

**Table 3:** Common method bias test.

Factor	Initial characteristic root			Extracted sum of squared loadings		
	Total	Variance %	Cumulative %	Total	Variance %	Cumulative %
1	6.836	31.073	31.073	6.836	31.073	31.073
2	2.480	11.271	42.344	2.480	11.271	42.344
3	2.240	10.181	52.525	2.240	10.181	52.525
4	1.715	7.797	60.322	1.715	7.797	60.322
5	1.340	6.092	66.413	1.340	6.092	66.413
6	1.135	5.158	71.571	1.135	5.158	71.571
7	1.004	4.563	76.134	1.004	4.563	76.134
8	0.955	4.339	80.473	-	-	-

### 3.4 Descriptive statistical analysis and correlation analysis

As shown in Table 4, correlation analysis showed positive correlations among growth mindset, career decision-making self-efficacy, perceived social support, and meaning in life. In addition, age and grade were positively correlated with career decision-making self-efficacy and meaning in life.

**Table 4:** Correlation analysis.

Variable	Age	Grade	GMS	CDSE	MSPSS
Age	1.000				
Grade	−0.057	1.000			
GMS	0.475**	0.472**	1.000		
CDSE	0.239**	0.247**	0.450**	1.000	
MSPSS	0.077	0.081	0.374**	0.359**	1.000
C-MLQ	0.306**	0.307**	0.538**	0.363**	0.374**

GMS, the Growth Mindset Scale; CDSE-SF, the Career Decision Self-Efficacy Scale–Short Form; MSPSS, the Multidimensional Scale of Perceived Social Support; C-MLQ, the Chinese version of the Meaning in Life Questionnaire. **P < 0.001.

### 3.5 Regression analysis of the mediating roles of perceived social support and meaning in life in the relationship between growth mindset and career decision-making self-efficacy

The Durbin–Watson statistic was used to test the independence of residuals. The Durbin–Watson value ranges from 0 to 4, with a value close to 2 indicating no autocorrelation. The results showed a Durbin–Watson value of 2.100, suggesting that there was no significant autocorrelation among the residuals and that the independence assumption of the regression analysis was satisfied. The Breusch–Pagan test yielded a *p*-value of 0.993, indicating insufficient evidence to suggest the presence of heteroscedasticity in the regression model. Multicollinearity among independent variables was assessed using the variance inflation factor (VIF). All VIF values were within the acceptable limits (typically < 10), indicating no serious multicollinearity. Overall, these results indicate that the data met the basic assumptions of linear regression analysis.

PROCESS Model 6 was used to conduct multivariate hierarchical regression analysis. As shown in Table 5, age and grade had no significant effects on career decision-making self-efficacy, indicating that the control variables had minimal influence on the primary relationships. Growth mindset had a significant direct effect on career decision-making self-efficacy (*β* = 1.761). Growth mindset positively predicted perceived social support (*β* = 1.278) and meaning in life (*β* = 0.978). Perceived social support positively predicted meaning in life (*β* = 0.170) and career decision-making self-efficacy (*β* = 0.433). Meaning in life also positively predicted career decision-making self-efficacy (*β* = 0.304).

**Table 5:** Regression analysis of the mediating roles of perceived social support and meaning in life in the relationship between growth mindset and career decision-making self-efficacy.

Regression equation		Overall fitting index			Regression coefficient	
Predictive variables	Outcome variables	R	R^2^	F	β	*t*
Perceived social support	Growth mindset	0.391	0.153	33.205	1.278	9.763**
	Age				−0.712	−0.976
	Grade				0.208	0.882
Meaning in life	Perceived social support	0.575	0.331	68.058	0.170	5.584**
	Growth mindset				0.978	9.625**
	Age				0.050	0.096
	Grade				0.600	0.597
Career decision-making self-efficacy	Perceived social support	0.508	0.258	38.328	0.433	4.992**
	Meaning in life				0.304	2.584*
	Growth mindset				1.761	5.801**
	Age				−0.907	−0.627
	Grade				2.858	1.028

Age and grade were used as control variables, *P < 0.05, **P < 0.001.

### 3.6 Analysis of the mediating effects of perceived social support and meaning in life on the relationship between growth mindset and career decision-making self-efficacy

**Figure 1:**
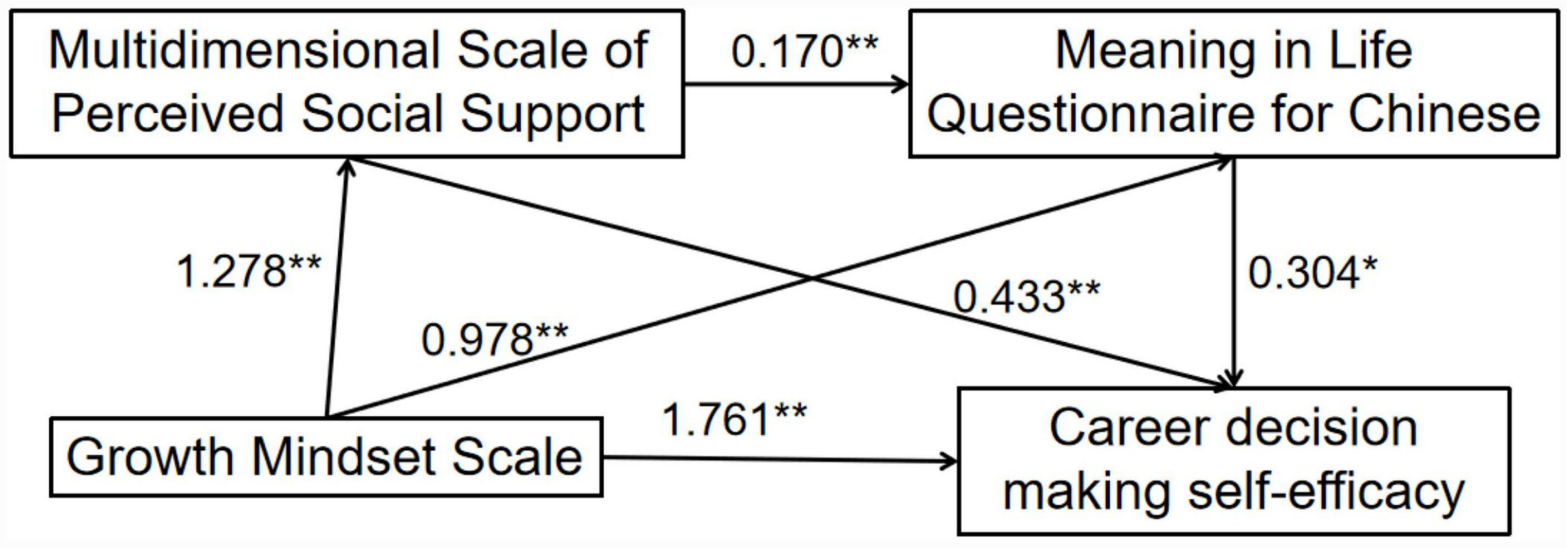
Chain mediation model of perceived social support and meaning in life in the relationship between growth mindset and career decision-making self-efficacy. *p < 0.05, **p < 0.001.

As shown in Table 6 and Figure 1, the bias-corrected bootstrap results indicated that the chain-mediating effect of perceived social support and meaning in life was significant, with a 95% confidence interval not including zero, accounting for 34.24% of the total effect. Among the three indirect paths, the effect sizes accounted for 20.65, 2.46, and 11.13% of the total effect.

**Table 6:** Analysis of the mediating effects of perceived social support and meaning in life on the relationship between growth mindset and career decision-making self-efficacy.

Route	Effect value	95%CI	Proportion
Direct effect	1.761	1.165–2.358	65.76%
Total intermediary effect	0.917	0.620–1.251	34.24%
GMS → MSPSS → CDSE-SF	0.553	0.349–0.794	20.65%
GMS → C-MLQ → CDSE-SF	0.066	0.019–0.145	2.46%
GMS → MSPSS → C-MLQ → CDSE-SF	0.298	0.084–0.551	11.13%

## 4 Discussion

Although the single-path relationship between growth mindset and career decision-making self-efficacy has been validated (Li et al., 2025; Cao et al., 2025), in the context of the ongoing challenging employment situation for college students in the post-pandemic era, identifying intervenable psychological resources and enhancing students’ job search confidence have become closely linked to national strategies for employment stabilization. This study is the first to integrate perceived social support and meaning in life into a chained-mediating model while focusing on students from traditional Chinese medicine universities, a group characterized by high professional barriers, unclear career paths, and an elevated need for psychological intervention. Compared to previous studies that examined only single mediators (Kwon and Lee, 2025), this study not only reveals the staged mechanism through which growth mindset operates—from external support to intrinsic meaning and then to career behavior—but also identifies actionable secondary intervention targets for university employment guidance.

The findings showed that growth mindset had a strong effect on career decision-making self-efficacy, with an effect value of 1.761, accounting for 65.76% of the total effect, thereby supporting Hypothesis I. This suggests that college students with a higher growth mindset are better able to enhance their self-efficacy and demonstrate greater confidence and engagement in the career decision-making process. In another study using a chain mediation approach, the direct effect of growth mindset on career decision-making self-efficacy was only 0.327, although it accounted for 71.09% of the total effect (Li et al., 2025). These findings suggest that the direct pathway from growth mindset to career decision-making self-efficacy consistently represents the core driving force, with indirect pathways playing a supplementary role. Multiple studies have demonstrated that growth mindset is significantly and positively associated with self-efficacy across various domains (Kondratowicz and Dorota Godlewska, 2022; Zhao et al., 2023; Girard et al., 2025). A strong growth mindset enhances college students’ confidence and enthusiasm, enabling them to actively confront challenges in career decision-making. Students with higher levels of growth mindset believe that sustained effort and accumulated experience can lead to success in a given career field and are, therefore, less likely to lose confidence or withdraw when facing challenges or uncertainty (Han, 2024; Harvey et al., 2025; He and Zhang, 2024).

The results also revealed a significant positive correlation between growth mindset and perceived social support, indicating that individuals with higher levels of growth mindset are more likely to perceive support from family, teachers, and peers. This finding is consistent with the study by Tamir et al. (2007), which showed that college students with stronger growth mindsets were better able to obtain social support from new friends and social networks. Further analysis indicated that the indirect pathway from growth mindset to career decision-making self-efficacy via perceived social support accounted for 20.65% of the total effect, supporting Hypothesis II. College students with a higher growth mindset are more capable of expanding their social networks, establishing broader social connections, and accessing richer support resources (Wang et al., 2021; Tao et al., 2022; Buddington, 2025). Moreover, interactions with diverse social groups facilitate access to employment-related information, and the accumulation of such information positively contributes to confidence and perceived competence in career decision-making (Zhang, 2024).

The findings further showed that growth mindset influenced career decision-making self-efficacy through the mediating role of meaning in life, with this indirect effect accounting for 2.46% of the total effect, thereby supporting Hypothesis III. Self-determination theory emphasizes that the satisfaction of the basic psychological needs for autonomy, competence, and relatedness is essential for motivation and personal growth (Flannery, 2017). Growth mindset primarily satisfies the need for competence, while the exploration and realization of meaning in life are closely related to autonomy and relatedness, together promoting the enhancement of career decision-making self-efficacy. Psychological resources theory posits that psychological resources such as hope, optimism, resilience, and self-efficacy strengthen individuals’ ability to cope with challenges. As a key psychological resource, growth mindset further enhances career decision-making self-efficacy by reinforcing individuals’ sense of meaning in life (Karras et al., 2022; Ommundsen et al., 2024; Chaudhry et al., 2024).

Finally, the results showed that perceived social support was positively associated with meaning in life, which is consistent with previous research (Zimet et al., 1990; Yuan et al., 2024). Moreover, perceived social support and meaning in life played a chain-mediating role in the relationship between growth mindset and career decision-making self-efficacy, accounting for 11.13% of the total effect and supporting Hypothesis IV. Growth mindset encourages individuals to adopt a positive attitude toward challenges. Through interaction, feedback, and encouragement from others, individuals receive emotional support and practical assistance, which enhances self-confidence and clarifies their career direction. Simultaneously, perceived social support helps individuals better recognize their values and potential during career decision-making, thereby strengthening their sense of meaning in life and confidence in future career development. As meaning in life increases, individuals adopt more positive perspectives on career choices and are better able to assess their ability to achieve goals during the decision-making process, ultimately enhancing their career decision-making self-efficacy.

## 5 Conclusion and future directions

Growth mindset had a significant direct effect on career decision-making self-efficacy (*β* = 1.76, accounting for 65.76% of the total effect), while the indirect pathway further contributed 34% through the chain of “perceived social support → meaning in life.” This study provides an in-depth examination of the mechanism through which growth mindset influences college students’ career decision-making self-efficacy, revealing the sequential mediating roles of perceived social support and meaning in life. Educational authorities may consider incorporating growth mindset interventions as an evidence-based strategy within employment stabilization policies, integrating growth mindset training into mandatory college career guidance curricula, establishing teacher certification systems and provincial data platforms, providing monthly subsidies of 300 yuan for students in high-risk majors such as traditional Chinese medicine through “growth mindset plus internship” programs, and conducting evaluations every 3 years to create a quantifiable and accountable policy tool for stabilizing employment.

Future research could expand the sample to include students from different regions and types of universities or conduct cross-cultural studies to verify the generalizability of the findings. In addition, longitudinal research designs could further elucidate the dynamic relationships among growth mindset, perceived social support, meaning in life, and career decision-making self-efficacy. Moreover, intervention studies focusing on growth mindset could offer more practical and operational solutions for university career guidance. Overall, this study not only advances theoretical research on the relationship between growth mindset and career decision-making self-efficacy but also offers practical guidance for universities in career counseling and mental health education.

## Data Availability

The original contributions presented in the study are included in the article/supplementary material, further inquiries can be directed to the corresponding authors.
